# Assessment of breast arteries and lymph nodes by 3D MR angiography enhancement imaging: feasibility and pilot clinical results

**DOI:** 10.1186/s12880-021-00629-w

**Published:** 2021-06-07

**Authors:** Yang Liu, Jiaxin Hou, Zhijun Zhu, Bingguang Liu, Manrui Cao, Wenjian Qin

**Affiliations:** 1 Affiliated Shenzhen Maternity & Child Healthcare Hospital, Southern Medical University, Shenzhen, 518028 People’s Republic of China; 2 Shenzhen Institute of Advanced Technology, Chinese Academy of Science, 1068 Xueyuan Avenue, Shenzhen, 518055 People’s Republic of China

**Keywords:** Magnetic resonance angiography, Breast disease, Lateral thoracic artery, Thoracodorsal artery, Lymph nodes

## Abstract

**Background:**

Conventional dynamic contrast enhanced (DCE) magnetic resonance (MR) hardly achieves a good imaging performance of arteries and lymph nodes in the breast area. Therefore, a new imaging method is needed for the assessment of breast arteries and lymph nodes.

**Methods:**

We performed prospective research. The research included 52 patients aged from 25 to 64 between June 2019 and April 2020. The isotropic e-THRIVE sequence scanned in the coronal direction after DCE-THRIVE. Reconstructed images obtained by DCE-THRIVE and the coronal e-THRIVE were compared mainly in terms of the completeness of the lateral thoracic artery, thoracodorsal artery, and lymph nodes. We proposed a criterion for evaluating image quality. According to the criterion, images were assigned a score from 1 to 5 according to the grade from low to high. Two board-certified doctors evaluated images individually, and their average score was taken as the final result. The chi-square test was used to assess the difference.

**Results:**

The coronal e-THRIVE score is 4.60, which is higher than the DCE-THRIVE score of 3.48, there are significant differences between the images obtained by two sequences (*P* = 1.2712e−8). According to the score of images, 44 patients (84.61%) had high-quality images on the bilateral breast. Only 3 patients’ (5.77%) images were not ideal on both sides. The improved method is effective for most patients to get better images.

**Conclusions:**

The proposed coronal e-THRIVE scan can get higher quality reconstruction images than the conventional method to visualize the course of arteries and the distribution of lymph nodes in most patients, which will be helpful for the clinical follow-up treatment.

## Background

Breast disease is a problem that has plagued women for a long time, such as breast cancer and some benign breast disease [1]. According to statistics, breast cancer has been the most common cancer worldwide with the highest incidence. In 2020, there are 2.3 million women diagnosed with breast cancer [2]. Benign breast disease also has the risk of progressing to breast cancer.

Imaging examination plays an important role in breast disease screening and clinical treatment guidance. Imaging can intuitively provide rich information that is of significance to clinical treatment, such as the course of vessels and distribution of lymph nodes. The axillary artery is one of the main blood supply arteries of the breast. The lateral thoracic artery and the thoracodorsal artery are branches of the axillary artery[3]. The blood supplement from the thoracodorsal artery to the breast is less than the lateral thoracic artery, while lymph nodes generally locate around the thoracodorsal artery [4]. It is important to identify the location of thoracodorsal arteries and lymph nodes in axillary lymph node dissection. During the operation, bleeding caused by the injury of the thoracodorsal artery and branches is necessary to avoid as far as possible [5]. Besides, for breast conserving surgery or breast repair surgery, the perforation and course of the thoracodorsal artery are also crucial bases for the thoracodorsal artery perforation flap [6].

Ultrasonic Doppler, computed tomography (CT) angiography, and magnetic resonance angiography (MRA) are the most common vascular imaging methods [7, 8]. Since Ultrasonic Doppler collects information segmentally with a time-consuming process, source vessels are unable to be traced [9]. Moreover, the quality of the ultrasonic Doppler examination is highly dependent on the professionalism of the operator. CT angiography inevitably exposes patients to ionizing radiation. Iodine contrast agents used for enhanced scanning may cause rare but well-defined anaphylaxis and nephrotoxicity [10]. However, MRA can more accurately display the number of perforating branch vessels, diameters, and vascular contorts in the subcutaneous adipose layer. These data provide an intuitive reference for preoperative design and intraoperative navigation [11]. The incidence of acute allergic reaction of gadolinium contrast agent used in enhanced scanning is much lower than that of iodine contrast agent, so the contrast agents used in MRA are safer and more stable [12]. Besides, Magnetic resonance (MR) is not only less demanding of the operator during the examination than ultrasound, but also allows retrospective diagnosis after the examination. However, the conventional breast MR has a low signal-to-noise ratio for the posterior part of the thorax, insufficient spatial discrimination, and limited scanning range, these result in a poor imaging quality and difficulties in 3D reconstruction. In particular, a lot of details are missing from the z-axis.

At present, there are few detailed reports on 3D MR angiography for breast arteries and lymph nodes, especially for guiding clinical operations, such as preparation of skin flaps. Based on the clinical significance and requirements of the lateral thoracic artery and thoracodorsal artery mentioned above, this paper proposed an improved MR scanning method for obtaining high-quality images, which significantly improved the image display integrity of the lateral thoracic artery, thoracodorsal artery and lymph nodes, to make the images play the guiding role better in clinical practice.

## Methods

### Patients

Between June 2019 and April 2020, 52 female patients who accepted breast MR imaging examination were enrolled in this study. The patients range from 25 to 64 years old, with an average age of 44.3. These patients including those who had breast mass and needed imaging diagnosis (n = 39), who had breast cancer and needed to observe treatment effect (n = 4), who had general breast disease except cancer and needed an examination (n = 7), and who took a physical examination (n = 2). The details of the patients are shown in Table 1. The inclusion criterion is as follows: patients who (1) were eligible for MR; (2) required plain scan and enhanced scan for breast MR. The exclusion criterion is as follows: patients who (1) had contraindications of MR; (2) had hepatic and renal insufficiency; (3) were allergic to the contrast agent. All the included patients had informed consent to the treatment plan and experiment contents, and they provided written informed consent before participation. This research had been approved by the ethics committee of the hospital.

**Table 1:** Characteristic of patients

*Age statistic*	
Age (y)	Case number
y ≤ 30	3
30 < y ≤ 40	15
40 < y ≤ 50	23
50 < y ≤ 60	10
y > 60	1
Total	52
*Clinical symptoms*	
Symptoms or needs	Case number
Breast mass	39
Breast cancer	4
Cyclomastopathy	3
Calcification	3
Occupying of breast duct	1
Physical examination (health)	2
Total	52

### Scan sequence and parameters

Philips Achieva 1.5 T dual-gradient MR imaging system was adopted with a switching rate of 122mT/m/ms and a gradient field of 33mT/m. The scan sequence was enhanced T1-weighted high resolution isotropic volume examination (e-THRIVE). The parameters of each sequence used in the examination are shown in Table 2.

**Table 2:** MRI parameters

Parameters	Plain scan				DCE-THRIVE (enhanced imaging)	3D coronal e-THRIVE
	T1-weighted imaging	T2-weighted imaging	Diffusion-weighted imaging	T2-weighted imaging		
Sequence	THRIVE	TSE	SE-EPI	TSE	dyn-THRIVE	e-THRIVE
Scan direction	Transverse	Transverse	Transverse	Transverse	Transverse	Coronal
Fold over direction	RL	RL	AP	RL	RL	RL
Fat suppression	None	None	SPAIR	SPAIR	SPAIR	SPAIR
TR/TE (ms)	8.4/4.6	3506/80	4442/47	3733/80	6.9/3.4	7.0/3.4
Flip angle (°)	12	90	90	90	12	12
Field of view (mm)	340 × 340	340 × 340	340 × 210	340 × 340	380 × 340	240 × 420
Voxel size (mm)	0.8 × 0.8	0.7 × 0.9	2.0 × 2.5	1.0 × 1.2	0.9 × 1.0	0.9 × 0.9 × 0.9
Slice thickness	3.5	3.5	5.0	3.5	2.0	0.9
No. of slices	80	40	24	40	85	180–210
TFE factor	40	18	43	19	47	46
Acquisition time (s)	99	98	102	134	45.5	133–154

DCE, dynamic contrast enhanced; THRIVE, T1-weighted high resolution isotropic volume examination; TSE, turbo spin echo; SE-EPI, spin-echo echo planar imaging; dyn-THRIVE, dynamic THRIVE; e-THRIVE, enhanced THRIVE; RL, right to left; AP, anterior to posterior; TR, repetition time; TE, echo time; SPAIR, spectral attenuated inversion recovery

Most conventional MR scans of the breast are transverse, which is hard to cover the entire breast area, resulting in loss of information about some lesions. Therefore, we used coronal scanning to increase the coverage on the feet to head (FH) direction to make the reconstructed image more complete. Setting fold over direction as right to left (RL) can avoid convolution artifacts caused by feet to head direction scan. Usually, artifacts are eliminated by adding a fold over suppression parameter, but the scanning time will increase. Due to the limitation of 8-channel coils, adopting sensitivity encoding (SENSE) to reduce the scanning time is not feasible. Therefore, by setting the coding direction to RL, we can avoid the influence of bilateral chest wall and armpit display caused by heartbeat. Meanwhile, using isotropic scanning is beneficial to the subsequent 3D reconstruction.

### Operating steps

**Fig. 1:**
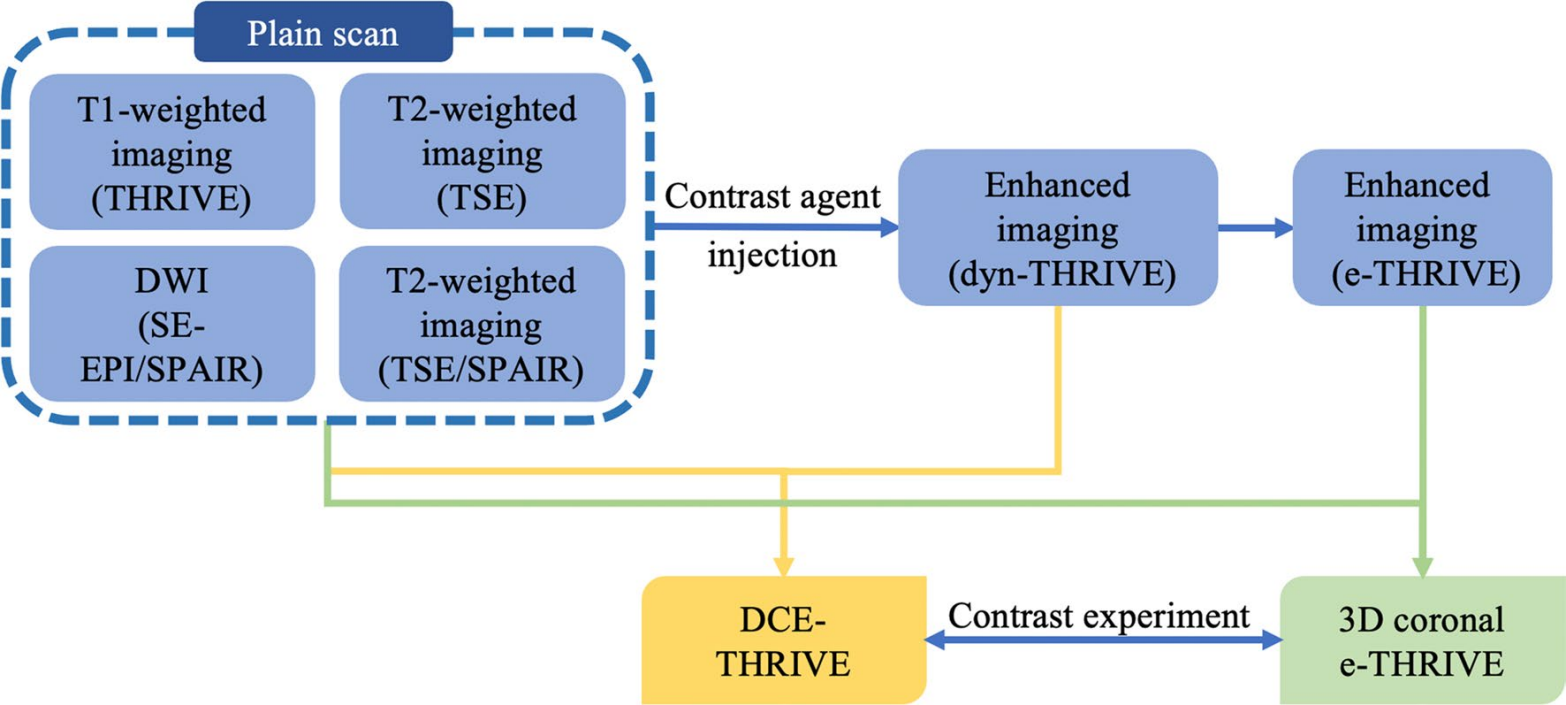
The operating steps of the scan. First, perform the plain scan of dynamic contrast enhancement MR, including T1-weighted imaging, T2-weighted imaging and diffusion weighted imaging, the sequences used in the scan written in the parentheses. Next, inject the contrast agent. Then, perform THRIVE sequence on the transverse direction and e-THRIVE sequence on the coronal direction. The reconstructed images of two sequences were compared

The scanning process is as follows: (1) Execute preparatory work for patients, remove all ferromagnetism and electronic products to ensure the safety of equipment and patients; (2) Puncture the median cubital vein and retain the indent needle, use a Nemoto SONIC shot GX high voltage syringe to inject 20 ml contrast agent (Gadolinium-diethylenetriamine pentaacetic acid, Gd-DTPA), the flow rate is 2.5 ml/s, and then flush the tube with 10 ml normal saline; (3) According to the scanning specification, the patient is required to lie prone on the coil, ensure to get the patient's full cooperation that not to move, turn on the positioning lamp; (4) Execute plain scan, and then, inject the contrast agent, execute breast dynamic sequence scan to complete dynamic contrast enhanced (DCE) THRIVE scan; (5) Execute e-THRIVE sequence on the coronal direction to complete 3D coronal e-THRIVE scan. The process is shown in Fig. 1.

### Data analysis

The data were imported into image reconstruction software (GE AW 4.6 workstation) and images were reconstructed by maximum intensity projection (MIP), volume rendering (VR), and multi-planar reconstruction (MPR). For DCE-THRIVE, because of the limitation of coverage area and anisotropy, images were reconstructed by MIP only in the transverse direction. For the coronal e-THRIVE, because the sequence is isotropic, images can be reconstructed by MIP in any direction. Thus, MIP image sets (18 projections separated by 10° along FH and RL axes) were generated for each patient. Moreover, to further observe the morphology of blood vessels and the distribution of lymph nodes, images also be reconstructed by VR and MPR for the coronal e-THRIVE. Each VR image set contained 18 images generated at 10° rotational increments along FH and RL axes. Each MPR image set contained images reconstructed along the longitudinal and transverse direction of blood vessels, which were used to measure the diameters of blood vessels.

To evaluate the images objectively, we had developed an evaluation criterion. The criterion divided the image quality into five grades. For each image, a score ranged from 1 to 5 was assigned according to the grade from low to high. The evaluation factors include lateral thoracic artery, thoracodorsal artery, and lymph nodes on the left side and right side. The details of the evaluation criterion are shown in Table 3. The average scores of arteries and lymph nodes were calculated to obtain a comprehensive evaluation of image quality. Two experienced physicians were invited (with 10 years of clinical experience) to assess images according to the criterion, and their average score was taken as the final result. In addition, we also designed a comparative experiment. The DCE-THRIVE reconstruction images of the same patients were evaluated using the above criteria and compared with the results of the new coronal e-THRIVE scan proposed in this paper.

**Table 3:** Evaluation criterion of imaging quality

Grade	Standard
5 (Excellent)	Clear display of vessels and their branches (complete and continuous); clear display of lymph nodes (quantity and distribution)
4 (Good)	Between grade 5 and grade 3
3 (General)	Partially display of vessels and branches (lost beginning or ending or display segmentally); partially display of lymph nodes
2 (Medium)	Between grade 3 and grade 1
1 (Poor)	Not display vessels and lymph nodes, affected by noise seriously

MIP image sets of DCE-THRIVE and the coronal e-THRIVE were used for the evaluation. A chi-square test was performed to evaluate the differences between the images obtained by two scan sequences [13]. A *p*-value of 0.05 or less was considered statistically significant. The above statistical analysis was performed by using IBM Statistical Product and Service Solutions 26.0 software.

## Results

**Fig. 2:**
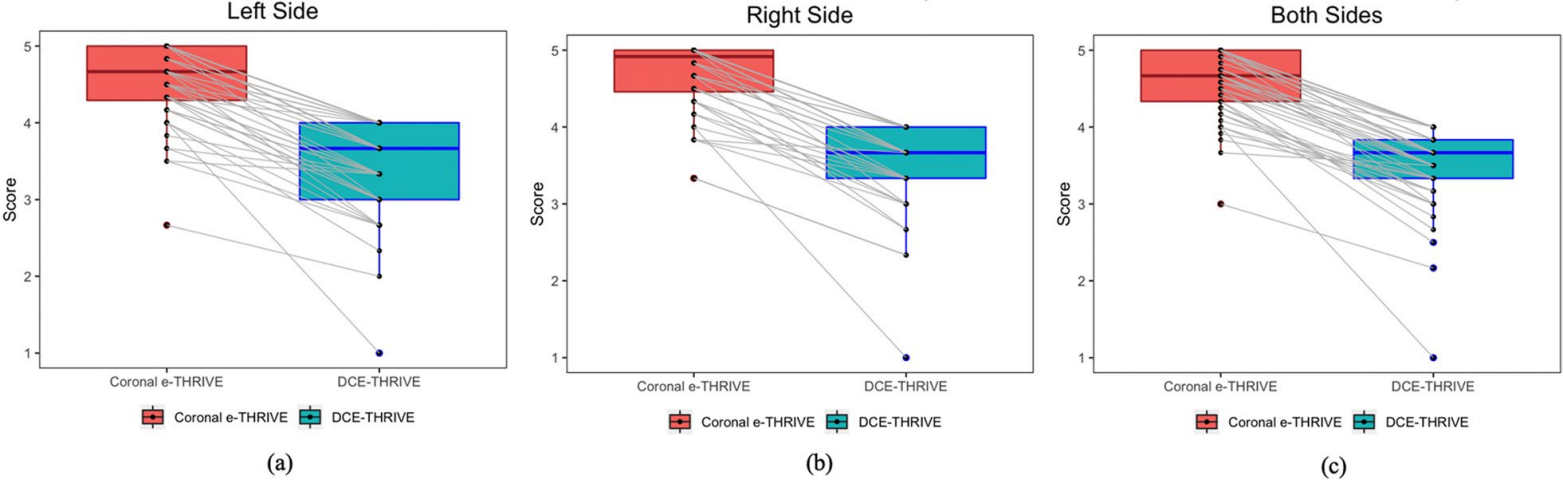
Assessment results comparison of images obtained by DCE-THRIVE and 3D coronal e-THRIVE. **a** Comparison on the left side. **b** Comparison on the right side. **c** Comparison on both sides

In total, 52 patients accepted examination, including the DCE-THRIVE scan and the coronal e-THRIVE scan. The scores assigned by two experienced physicians were collected and analyzed to demonstrated the quality of the coronal e-THRIVE scan. The details of the analysis results were shown in Table 4. For reconstructed images obtained by DCE-THRIVE, the average score of arteries and lymph nodes of all patients is 3.48, while that of the coronal e-THRIVE scan is 4.60. According to the chi-square test, the qualities of images obtained by two scan sequences are significantly different, whether the arteries and lymph nodes assessment or the comprehensive assessment (*P* = 1.2712e−8). The difference is intuitive from the boxplots in Fig. 2.

**Table 4:** Evaluation results of image quality of DCE-THRIVE and the coronal e-THRIVE scan

Objects	DCE-THRIVE	Coronal e-THRIVE	*P* value
*Left*			
Lateral thoracic artery	3.67 ± 0.76 (1.0–4.0)	4.61 ± 0.81 (1.0–5.0)	9.9945e−15
Thoracodorsal artery	3.04 ± 1.05 (1.0–4.0)	4.10 ± 0.87 (1.0–5.0)	2.5398e−11
Lymph nodes	3.60 ± 0.75 (1.0–4.0)	4.91 ± 0.39 (1.0–5.0)	1.9898e−18
*Right*			
Lateral thoracic artery	3.71 ± 0.78 (1.0–4.0)	4.60 ± 0.77 (1.0–5.0)	7.4878e−15
Thoracodorsal artery	3.33 ± 0.94 (1.0–4.0)	4.46 ± 0.79 (1.0–5.0)	9.5572e−13
Lymph nodes	3.56 ± 0.75 (1.0–4.0)	4.92 ± 0.39 (1.0–5.0)	3.4704e−19
Comprehensive assessment	3.48 ± 0.55 (1.0–4.0)	4.60 ± 0.44 (3.0–5.0)	1.2712e−8

Data are means and standard deviation. The scoring range of 2 doctors are shown in parentheses

*P* < 0.05 was considered statistically significant

**Fig. 3:**
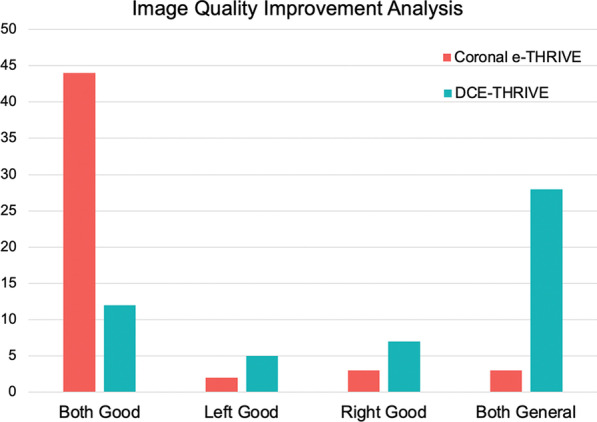
Comparison of breast image quality of each patient on the left side and right side

The reconstructed image score of each case was then calculated. Among the 52 patients, 44 of them (84.61%) had obtained high-quality images of arteries and lymph nodes on both sides (the average scores of both sides were higher than 4.0). 3 patients’ (5.77%) images quality were slightly inferior on the left side (the score of the left side was lower than 4.0, while that of the right side was higher than 4.0). 2 patients’ (3.85%) images quality were slightly inferior on the right side (the score of the right side was lower than 4.0, while that of the right side was lower than 4.0)0.3 patients’ (5.77%) images on both sides were not ideal (the scores of both sides were lower than 4.0). By applying coronal e-THRIVE scan, the number of patients with good bilateral image quality increased significantly, while the number of patients with general bilateral image quality decreases. The details of the statistic were shown in Table 5. The statistical comparison of the number of patients who obtained high-quality images by DCE-THRIVE and coronal e-THRIVE was shown in Fig. 3.

**Table 5:** Comparison of comprehensive assessment of image quality between DCE-THRIVE and the coronal e-THRIVE scan

Grading range (average)	Case number (percentage)	
	DCE-THRIVE	Coronal e-THRIVE
Left side good (≥ 4.0) Right side good (≥ 4.0)	12 (23.07%)	44 (84.61%)
Left side good (≥ 4.0) Right side general (< 4.0)	5 (9.62%)	2 (3.85%)
Left side general (< 4.0) Right side good (≥ 4.0)	7 (13.46%)	3 (5.77%)
Left side general (< 4.0) Right side general (< 4.0)	28 (53.85%)	3 (5.77%)

**Fig. 4:**
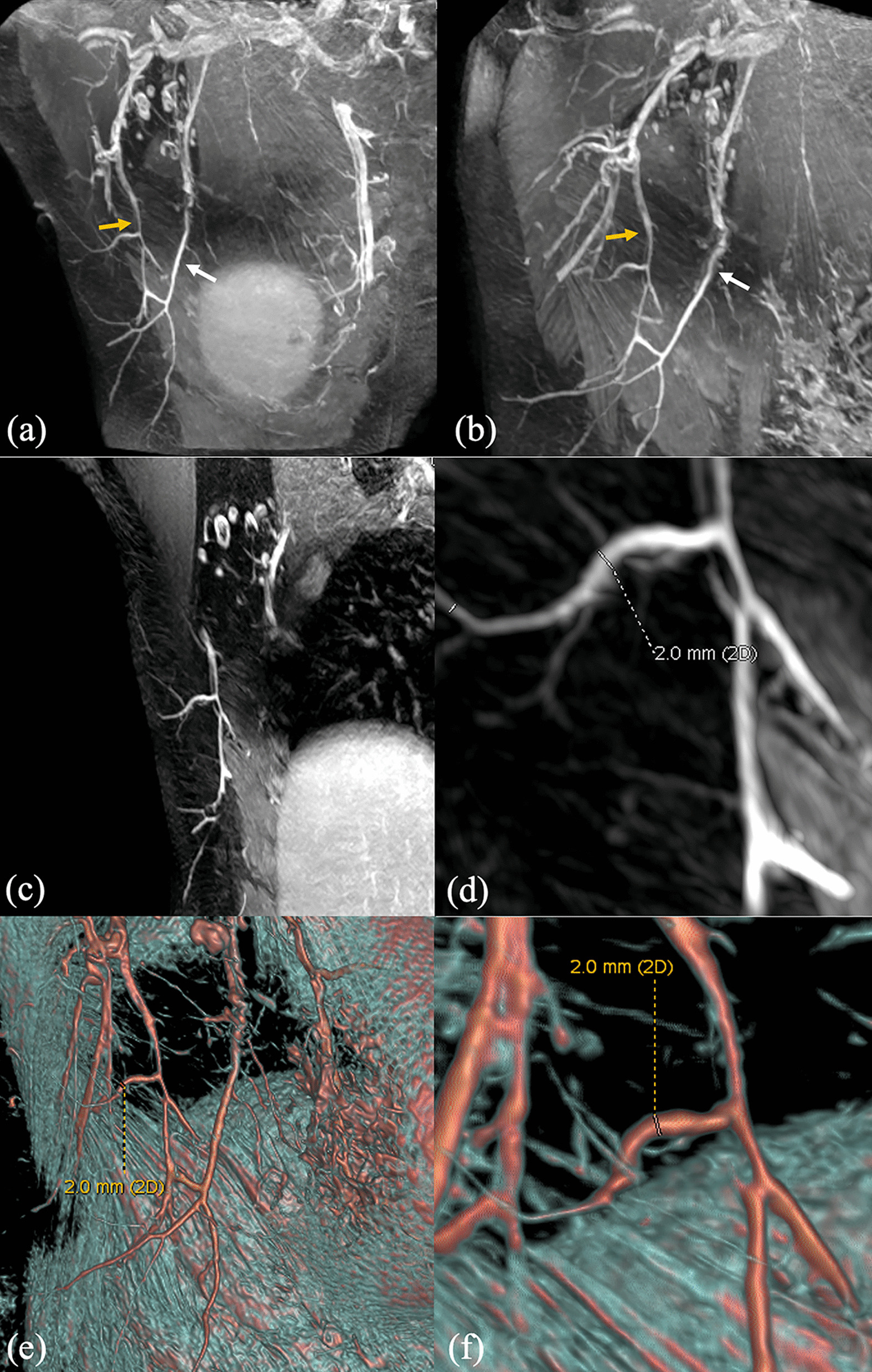
Maximum intensity projection (MIP) images, multi-planar reconstruction (MPR) images, and volume rendering (VR) images obtained by 3D coronal e-THRIVE. All images display lateral thoracic artery and thoracodorsal artery of the right breast of a 44-year-old patient. **a**, **b** MIP images of two different angles. **c**, **d** MPR images. Zoom in on (**c**) to get (**d**). **e**, **f** are VR images from the reconstructed 3D model. Zoom in on (**e**) to get (**f**)

**Fig. 5:**
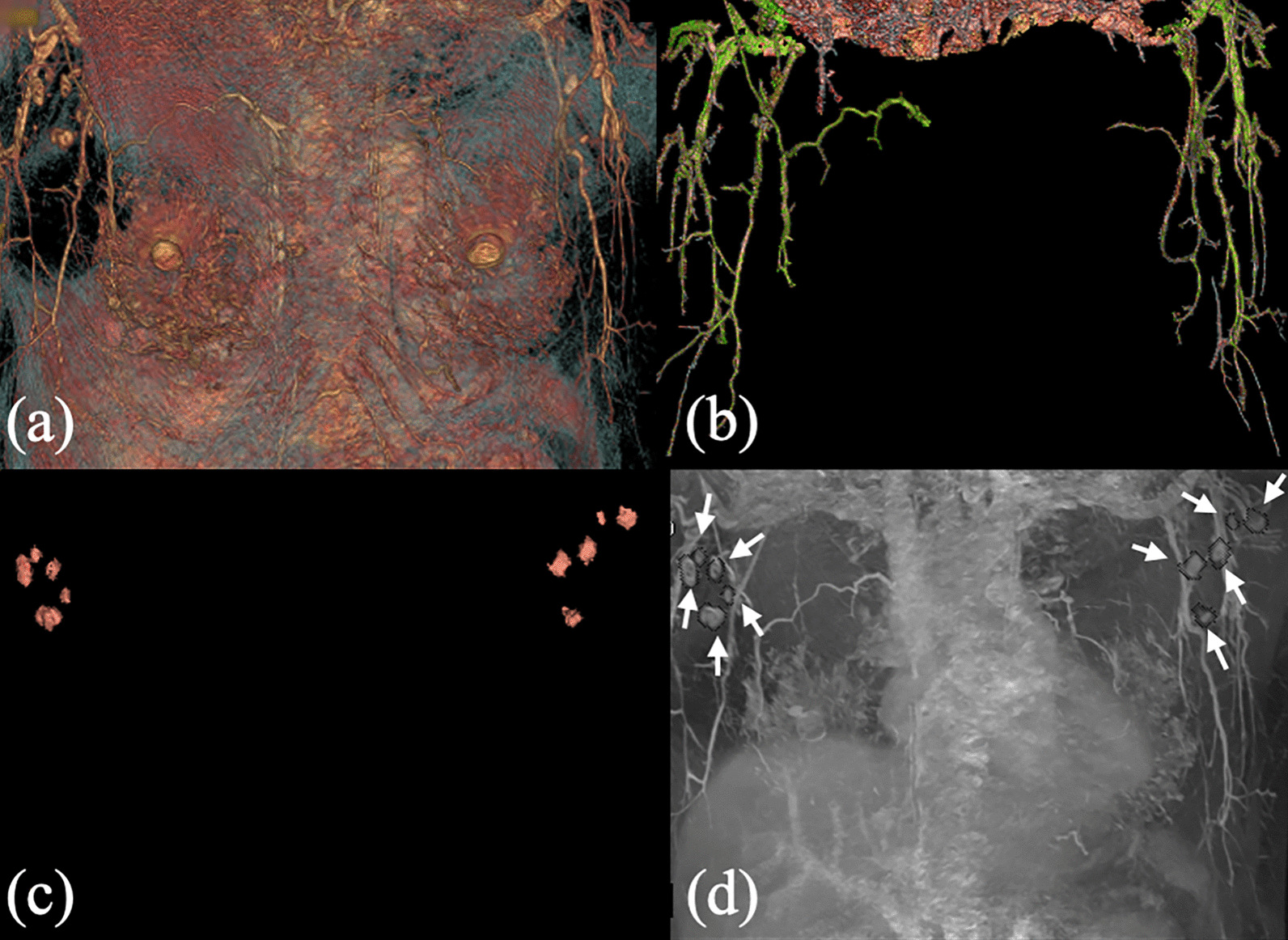
**a** Volume rendering (VR) image of the lateral thoracic artery and thoracodorsal artery. **b** Breast bilateral arteries extracted from the VR 3D model. **c** Lymph nodes extracted from the VR 3D model. **d** Maximum intensity projection image of the same case of **a**. Lymph nodes image extracted from **a** can be used to enhance the image of lymph nodes in (d). The white arrows indicate lymph nodes

We selected some representative reconstructed images of patients to show effectiveness on quality improvement by performing the coronal e-THRIVE scan. In Fig. 4, MIP, VR, and MPR images on the breast right side of a 44-year-old patient were shown. These images displayed lateral thoracic artery and thoracodorsal artery. Figure 4a, b were MIP images reconstructed from different angles showed the morphology of the thoracodorsal artery (indicated by yellow arrows), lateral thoracic artery (indicated by white arrows) and their branches completely. Figure 4c, d were MPR images generated in the direction of longitudinal of the arteries. The diameters of the arteries could measure in the images. For the case in Fig. 4, the diameters of the thoracodorsal artery branch range from 1.5 to 2.0 mm, and peripheral branches range from 0.8 to 1.0 mm. Figure 4e, f showed the model reconstructed by VR, which displayed the location of blood arteries and distribution of lymph nodes more intuitively. VR can also display arteries or lymph nodes separately, as shown in Fig. 5. This function made it easier to observe the morphology and branches of blood arteries without the interference of surrounding tissues. The combination of the three kinds of images can obtain more comprehensive information on the morphology, quantity, and location of arteries and lymph nodes. For example, as shown in Fig. 5d, we used the extracted lymph nodes from the VR model to enhance its effect in MIP images. In summary, various reconstructed images can be combined to play a better role in clinical diagnosis and treatment.

## Discussion

In this paper, we proposed an improved scan method of MRA, which displays lateral thoracic artery, thoracodorsal artery, and lymph nodes clearly and completely. Based on conventional DCE-THRIVE, the e-THRIVE sequence was added in coronal direction instead of transverse. After the original scanning data were obtained, we reconstructed them by MIP, MPR, VR to reflect the course of arteries and the situation of lymph nodes from various perspectives. Through reasonable statistical analysis, we had proved that the image quality of the coronal e-THRIVE sequence is superior to that of the conventional DCE-THRIVE scan, especially on displaying arteries and lymph nodes. The coronal e-THRIVE scan is feasible and has a clinical reference value. Meanwhile, these scanning had lower requirements for equipment, while the results improved significantly. It reduces the cost of imaging examination for patients and makes it easier to popularize MRA in breast screening.

Image diagnosis is one of the common methods for direct and rapid diagnosis of disease [14]. In MRA studies, the cardiovascular [15], lower extremities [16], cerebrovascular [17], carotid artery [18], etc. are the major research work. However, there are few studies on breast angiography, and the imaging of breast vessels and their branches is of great help to the diagnosis of breast diseases [19]. Vessels can also be used for surgical treatment [20]. Petrillo A. et al. [21] used contrast-enhanced MRI and a semi-automatic procedure to obtain a breast vascular map and evaluated the correlation between vessels and tumor location. Vasile et al. [22] located the position of vessels by using MRA. The position is useful for the surgery of perforator flap breast reconstruction. The imaging methods used in these studies are all transverse imaging. However, there is a problem in practice that the z-axis scan range is limited, resulting in the loss of detailed information. We conducted a coronary imaging study that showed a significant improvement in the completeness of breast artery information capture compared to conventional methods.

**Fig. 6:**
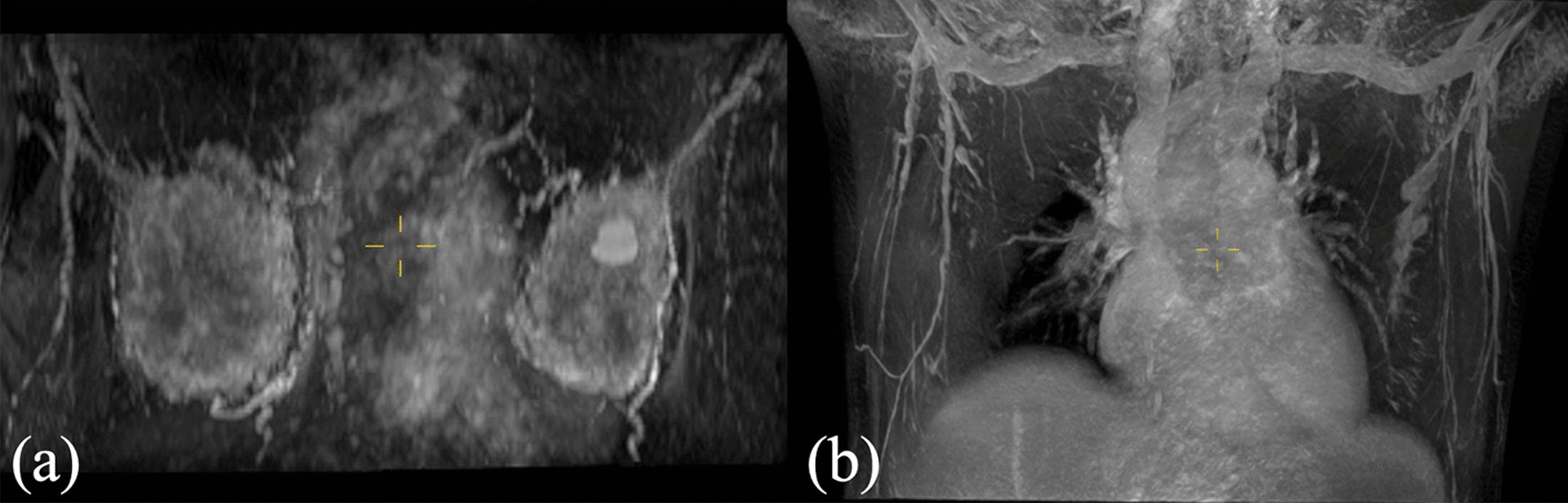
**a** The reconstructed image of an arterial phase in the process of DCE-THRIVE, which voxel size is 0.9 × 0.9 × 2.0 mm; **b** the reconstructed image of coronal e-THRIVE scan which voxel size is 0.9 × 0.9 × 0.9 mm

The conventional methods are usually transverse scanning and dynamic sequence is used to obtain the original data after plain scanning. In order to obtain better temporal resolution, the dynamic enhancement sequence should be coordinated with breast wash in and out. During the scanning process, one phase image is generated every minute. The problems of the method are as follows: the thickness of the layer is too large, which brings difficulties to the later 3D reconstruction; the coverage area in the direction from head to feet is small, usually around 170 mm, which cannot cover the beginning of arteries and the ending of branches. If the coverage area expands by increasing the number of layers, the scanning time will be too long. It brings pain to patients. If the coverage area expands by increasing the layer thickness, the resolution of the z-axis will reduce again. Whether the thickness is too large or the coverage area is too small, the information of the breast area will lose, resulting in a false-negative diagnosis, which harms the health of the patients. By adding the coronal e-THRIVE scan mentioned in this paper, the spatial resolution is improved to 0.9 × 0.9 × 0.9 mm, covering a range of about 240 mm on the z-axis of the human body with an appropriate signal-to-noise ratio. We chose an arterial phase to reconstruct Fig. 6a in the process of DCE-THRIVE. Then, in the process of coronal e-THRIVE of the same patient, we reconstruct Fig. 6b of the same area. It can be seen that coronal e-THRIVE is better in the reconstruction of coronal plane images for arteries and lymph nodes display.

In clinical practice, 16 channels MR scanning machines, which have large magnetic fields are common. These types of equipment can get high-quality images. While the proposed improved method can obtain equal image quality by 1.5 T machine with 8 channels, which is of great significance to reduce the cost of examination and promote the popularization of MRA examination. In addition, the coronal e-THRIVE sequence was added after the routine DCE-THRIVE to combine routine breast examination with angiography. Patients were injected with contrast agent only one time. They can get high-quality images of breast arteries and lymph nodes while completing the routine examination. On the one hand, it will not increase the economic burden of patients; the dosage of contrast agents will not increase, which ensures the safety of patients and reduces the pain and health burden of patients. For hospitals and doctors, it also improves work efficiency.

### Limitations

In addition, this method also has some shortcomings. The main problem is that the scanning time will increase about 2 min compared with DCE-THRIVE because of adding the coronal e-THRIVE sequence. The extension of time may cause pain for some patients. The process of image reconstruction may be influenced by noise from outside or caused by the reconstruction method itself, which leads to image blurring or inaccurate [23]. For accurate measurements, these effects should be removed by preprocessing [24–26], which should be considered in the future of work. And the other problem is that the research results in this paper are mainly limited to the acquisition of morphology of lateral thoracic artery, thoracodorsal artery, and lymph nodes, and there is a lack of quantitative analysis in terms of location and quantity, which need more clinical practice not just imaging analysis. Besides, some other factors may affect the image quality, such as (1) the movement of patients caused by too long time and uncomfortable position; (2) motion artifact caused by heartbeat and breathing; (3) the main magnetic field is uneven caused by poor equipment state, and leading to the poor effect of fat suppression which affects the display of arteries; (4) the problems of blood circulation of patients; (5) the dosage of contrast agent is not suitable. In summary, the technique of breast MRA still has some room for improvement.

## Conclusions

In conclusion, the proposed coronal e-THRIVE scan can get higher quality reconstruction images than the conventional methods, and the effectiveness and feasibility of the improved method in the assessment of breast arteries and lymph nodes were prospectively studied. Finally, a quantitative comparison experiments has been performed by experienced physicians. In our next research, we will continue to solve the problems mentioned above. It is necessary to reduce the scanning time. Furthermore, we will explore imaging methods that can show more vessels of the breast, which will be helpful for the clinical follow-up treatment.

## Data Availability

The datasets analyzed during the current study are available from the corresponding author on reasonable request.

## Abbreviations

AP: Anterior to posterior
CT: Computed Tomography
DCE: Dynamic contrast enhanced
Gd-DTPA: Gadolinium-diethylenetriamine pentaacetic acid
MR: Magnetic resonance
MRA: Magnetic resonance angiography
MIP: Maximum intensity projection
MPR: Multi-planar reconstruction
RL: Right to left
SE-EPI: Spin-echo echo planar imaging
SENSE: Sensitivity encoding
SPAIR: Spectral attenuated inversion recovery
TE: Echo time
THRIVE: T1-weighted high resolution isotropic volume examination
dyn-THRIVE: Dynamic T1-weighted high resolution isotropic volume examination
e-THRIVE: Enhanced T1-weighted high resolution isotropic volume examination
TR: Repetition time
TSE: Turbo spin echo
VR: Volume rendering

## References

[1] Lynn CH, Thomas AS, Marlene HF, Wilma LL, Amy CD, Karthik G (2005). Benign breast disease and the risk of breast cancer. N Engl J Med.

[2] Sung H, Ferlay J, Siegel RL, Laversanne M, Soerjomataram I, Jemal A (2021). Global cancer statistics 2020: GLOBOCAN estimates of incidence and mortality worldwide for 36 cancers in 185 countries. CA Cancer J Clin.

[3] Loukas M, Plessis M, Owens DG, Kinsella CR, Litchfield CR, Nacar A (2014). The lateral thoracic artery revisited. Surg Radiol Anat.

[4] Heitmann C, Guerra A, Metzinger SW, Levin LS, Allen RJ (2003). The thoracodorsal artery perforator flap: anatomic basis and clinical application. Ann Plast Surg.

[5] Veronesi U, Paganelli G, Galimberti V, Viale G, Zurrida S, Bedoni M (1997). Sentinel-node biopsy to avoid axillary dissection in breast cancer with clinically negative lymph-nodes. Lancet.

[6] Ojeda ABL, Lopez CC, Rodrigues TAG, Vidal JM, Suñe CH, Segu JOB (2013). Thoracodorsal artery perforator (TDAP) flap in immediate breast reconstruction and the role of preoperative mapping: a clinical experience. Eur J Plast Surg.

[7] Shen MH, Abulaiti A, Yushufu A, Dong J, Wang W, Liu YF (2014). Thoracic dorsal artery perforating flap transplantation under high-frequency ultrasound guidance. Chin J Tissue Eng Res.

[8] Shen MH, Ren P, Xiao H, Yusuful A, Yakufu M, Wang Y (2015). Three-dimensional visual research of thoracic dorsal artery based on CT angiography. Chin J Reparative Reconstr Surg.

[9] Lin CT, Huang JS, Hsu KC, Yang KC, Chen JS, Chen LW (2008). Different types of suprafascial courses in thoracodorsal artery skin perforators. Plast Reconstr Surg.

[10] Parfrey P (2005). The clinical epidemiology of contrast-induced nephropathy. Cardio Vasc Interv Radiol.

[11] Runge VM, Kirsch JE, Lee C (1993). Contrast-enhanced MR angiography. J Magn Reson Imaging.

[12] Sonoda A, Nitta N, Tsuchiya K, Nitta-Seko A, Ohta S, Otani H (2016). A novel blood-pooling MR contrast agent: carboxymethyl-diethylaminoethyl dextran magnetite. Mol Med Rep.

[13] Cochran WG (1954). Some methods for strengthening the common χ^2^ tests. Biometrics.

[14] Houssami N, Hayes DF (2009). Review of preoperative magnetic resonance imaging (MRI) in breast cancer: should MRI be performed on all women with newly diagnosed, early stage breast cancer?. CA Cancer J Clin.

[15] Luo J, Addy NO, Ingle RR, Baron CA, Cheng JY, Hu BS, Nishimura DG (2017). Nonrigid motion correction with 3D image-based navigators for coronary MR angiography. Magn Reson Med.

[16] Mohrs OK, Petersen SE, Heidt MC, Schulze T, Schmitt P, Bergemann S (2011). High-resolution 3D non-contrast-enhanced, ECG-gated, multi-step MR angiography of the lower extremities: comparison with contrast-enhanced MR angiography. Eur Radiol.

[17] Krishnan GH, Umashankar G, Abraham S (2016). Cerebrovascular disorder diagnosis using MR angiography. Biomed Res.

[18] Koktzoglou I, Giri S, Piccini D, Grodzki DM, Flanagan O, Murphy IG (2015). Arterial spin labeled carotid MR angiography: a phantom study examining the impact of technical and hemodynamic factors. Magn Reson Med.

[19] Onishi N, Kataoka M, Kanao S, Sagawa H, Iima M, Nickel MD (2018). Ultrafast dynamic contrast-enhanced MRI of the breast using compressed sensing: breast cancer diagnosis based on separate visualization of breast arteries and veins. J Magn Reson Imaging.

[20] Chong LW, Lakshminarayan R, Akali A (2019). Utilisation of contrast-enhanced magnetic resonance angiography in the assessment of deep inferior epigastric artery perforator flap for breast reconstruction surgery. Clin Radiol.

[21] Petrillo A, Fusco R, Filice S, Granata V, Catalano O, Vallone P (2016). Breast contrast enhanced MR imaging: semi-automatic detection of vascular map and predominant feeding vessel. PLoS ONE.

[22] Vasile JV, Levine JL (2016). Magnetic resonance angiography in perforator flap breast reconstruction. Gland Surg.

[23] Toğaçar M, Ergen B, Cömert Z (2020). COVID-19 detection using deep learning models to exploit Social Mimic Optimization and structured chest X-ray images using fuzzy color and stacking approaches. Comput Biol Med.

[24] Versaci M, Morabito FC, Angiulli G (2017). Adaptive image contrast enhancement by computing distances into a 4-dimensional fuzzy unit hypercube. IEEE Access.

[25] Rahim SS, Palade V, Shuttleworth J, Jayne C (2016). Automatic screening and classification of diabetic retinopathy and maculopathy using fuzzy image processing. Brain Inform.

[26] Versaci M, Morabito FC (2021). Image edge detection: a new approach based on fuzzy entropy and fuzzy divergence. Int J Fuzzy Syst.

